# Subcutaneous Lanreotide Depot: A Diagnostic Pitfall in Neuroendocrine Tumor Imaging

**DOI:** 10.7759/cureus.79436

**Published:** 2025-02-22

**Authors:** Tsutomu Nishida

**Affiliations:** 1 Department of Gastroenterology, Toyonaka Municipal Hospital, Toyonaka, JPN

**Keywords:** computed tomography, ct imaging, lanreotide, neuroendocrine tumor, octreoscan, somatostatin analog

## Abstract

Lanreotide depot, a long-acting somatostatin analog, improves progression-free survival in neuroendocrine tumor (NET) patients. However, persistent subcutaneous depots can appear as metastatic lesions on imaging, potentially leading to misdiagnosis. We report the case of a 70-year-old man with grade 2 NET (Ki-67: 5.9%) undergoing six years of lanreotide treatment. Follow-up imaging revealed stable primary disease. However, nodular lesions in the buttock were identified on the CT scan performed eight months after the start of treatment. A retrospective review of a CT scan taken two months after treatment initiation revealed that these nodules were already present and had gradually increased in number, allowing for their correct identification as persistent lanreotide depots. This case underscores the need for heightened diagnostic awareness to prevent unnecessary interventions and ensure accurate management.

## Introduction

Neuroendocrine tumors (NETs) are a diverse group of neoplasms originating from neuroendocrine cells distributed throughout various organs in the body. NETs are classified based on their functional status (hormone-secreting vs. non-secreting) and proliferative activity, as indicated by the Ki-67 index according to the WHO classification (G1: <3%, G2: 3-20%, G3: >20%) [1]. Their management includes surgical resection, targeted therapies, peptide receptor radionuclide therapy [2,3], and somatostatin analogs such as octreotide and lanreotide [4-6]. Among these treatments, lanreotide depot has shown significant efficacy in prolonging progression-free survival in patients with well-differentiated, unresectable, or metastatic NETs, as demonstrated in the CLARINET trial [7,8]. However, despite its clinical efficacy, the radiological characteristics of lanreotide depots are often overlooked, which may lead to diagnostic pitfalls. Specifically, residual depots at subcutaneous injection sites can be mistaken for metastatic lesions, potentially triggering unnecessary diagnostic interventions and causing patient anxiety [9].

This case report highlights the importance of recognizing the imaging characteristics of lanreotide depots to avoid misdiagnosis and ensure appropriate clinical management. Recognizing this phenomenon can help healthcare providers prevent diagnostic errors and optimize patient management.

## Case presentation

**Figure 1:**
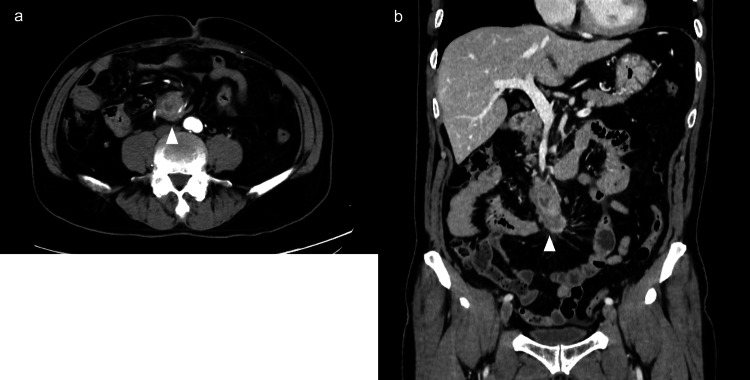
Contrast-enhanced CT images Contrast-enhanced CT imaging showing an irregularly shaped mass measuring 30 × 50 mm in the mesentery near the duodenum, along with several small lymph nodes in the surrounding area (a: axial view; b: coronal view). CT, computed tomography

A 70-year-old man with a history of coronary artery disease was referred for evaluation following an incidental detection of suspected para-aortic lymphadenopathy on routine echocardiography. Contrast-enhanced CT imaging showed an irregularly shaped mass measuring 30 × 50 mm in the mesentery near the duodenum, along with several small lymph nodes in the surrounding area (Figures 1a, 1b).

A definitive diagnosis could not be made based on imaging alone. Differential diagnosis included gastrointestinal stromal tumor originating from the duodenum, malignant lymphoma, mesenteric fibromatosis, solitary fibrous tumor, and metastatic NET.

Tumor marker analysis revealed normal levels of carcinoembryonic antigen (CEA) at 1.4 ng/mL (reference: 0-5 ng/mL), carbohydrate antigen 19-9 (CA19-9) at 5 U/mL (reference: <37 U/mL), and soluble interleukin-2 receptor (sIL-2R) at 446 U/mL (reference: 156.6-474 U/mL), all within their respective reference ranges. The interferon-gamma release assay for tuberculosis yielded negative results. Esophagogastroduodenoscopy and colonoscopy did not reveal any obvious malignant lesions.

**Figure 2:**
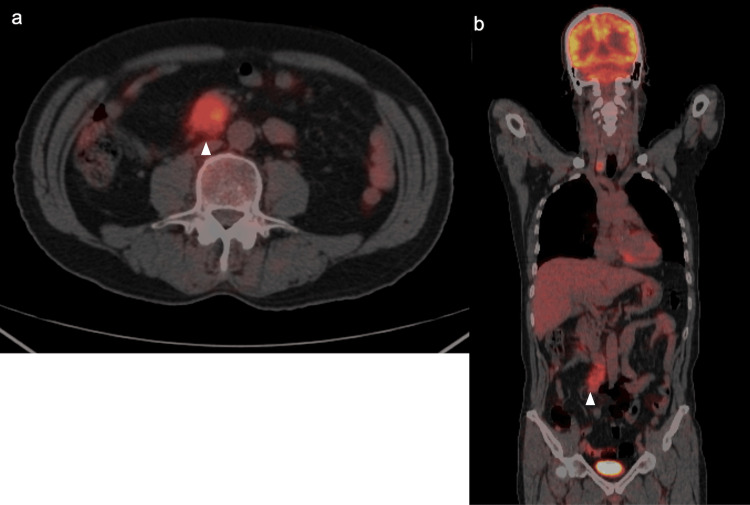
FDG-PET CT scan FDG-PET CT revealed FDG uptake with an SUVmax of 5.1 in the same lesion (a: axial view; b: coronal view). Arrows indicate tumor locations. FDG-PET, fluorodeoxyglucose positron emission tomography; CT, computed tomography

Fluorodeoxyglucose positron emission tomography (FDG-PET) CT scan detected the same lesion, with a maximum standardized uptake value (SUVmax) of 5.1 (Figures 2a, 2b).

**Figure 3:**
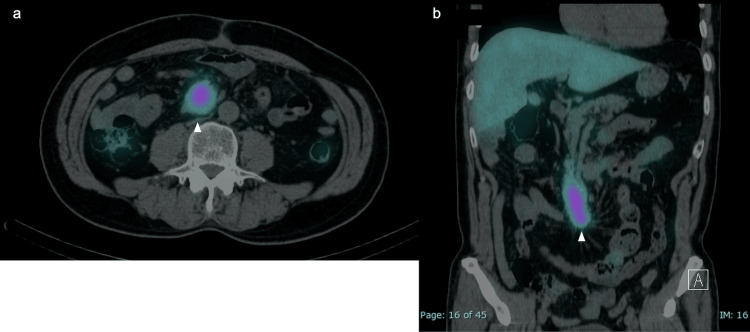
OctreoScan images OctreoScan images showing neuroendocrine tumors of the small intestine (a: axial view; b: coronal view). Arrows indicate tumor locations.

OctreoScan imaging revealed positive findings with a high uptake of radiolabeled somatostatin, consistent with a tumor (Figures 3a, 3b).

Endoscopic ultrasound-guided fine-needle aspiration was attempted; however, an adequate sample could not be obtained. This was likely due to the anatomical location of the tumor, which made successful tissue acquisition challenging. Consequently, surgical resection was initially considered, but intraoperative findings revealed arterial invasion, rendering complete resection unfeasible. Therefore, a tumor biopsy was performed to establish a definitive diagnosis. During the laparotomy, no ascites or peritoneal dissemination was observed. A 5-cm white, elastic, soft mass was identified in the mesentery of the small intestine near the superior mesenteric vein. Histopathological examination confirmed the diagnosis of a grade 2 NET, with a Ki-67 index of 5.9%. Immunohistochemical analysis further supported this diagnosis, with positive staining for synaptophysin, chromogranin A, and CD56, along with mitotic figures observed at 1 per 10 high-power fields (HPF).

Following this diagnosis, treatment with subcutaneous lanreotide depot injections (120 mg every four weeks) was initiated in May 2019. At the start of the treatment, the neuron-specific enolase level was 9.5 ng/mL (reference: <16.3 ng/mL) and remained stable. Gastrin levels were mildly elevated at 245 pg/mL (reference: 37-172 pg/mL) and immuno-reactive insulin at 21.6 μU/mL (reference: 1.7-10.4 μU/mL), but there were no hormone symptoms, and we suspected the effects of proton pump inhibitor medication and insulin resistance. There was also no increase in serum calcium levels.

**Figure 4:**
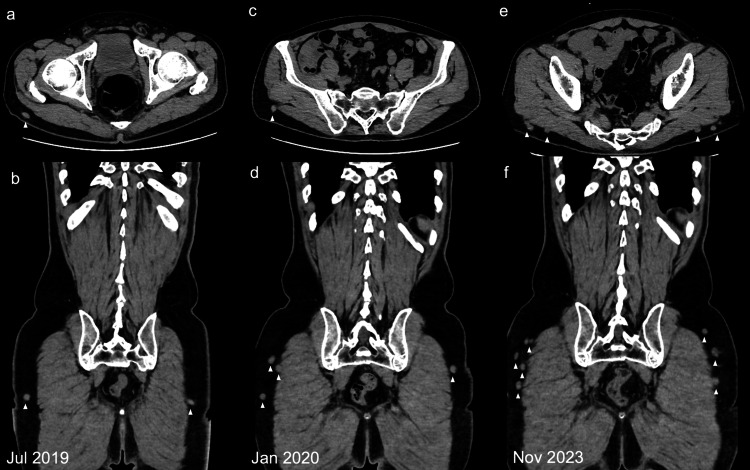
Follow-up CT images Follow-up C) images after 3 (a, b), 10 (c, d), and 60 (e, f) lanreotide depot injections (a, c, e: axial views; b, d, f: coronal views). Persistent nodular lesions (arrows) in subcutaneous nodules on the buttocks over time. CT, computed tomography

On follow-up CT scans over a period of six years (72 doses), the primary tumor site was stable; however, nodular lesions in the buttock were identified on the CT scan performed eight months after the start of treatment (Figures 4c, 4d). A retrospective review of previous CT scans revealed that these nodules were present on the July 2019 scan two months after the start of treatment (Figures 4a, 4b) and gradually increased thereafter (Figures 4e, 4g). Initially, the possibility of recurrence was considered.

However, based on the clinical course and previously reported cases, the nodules were determined to be unrelated to metastasis. As a result, follow-up PET-CT and OctreoScan imaging were not performed. Despite their presence, the patient remained asymptomatic, and the nodules were not palpable on physical examination.

**Figure 5:**
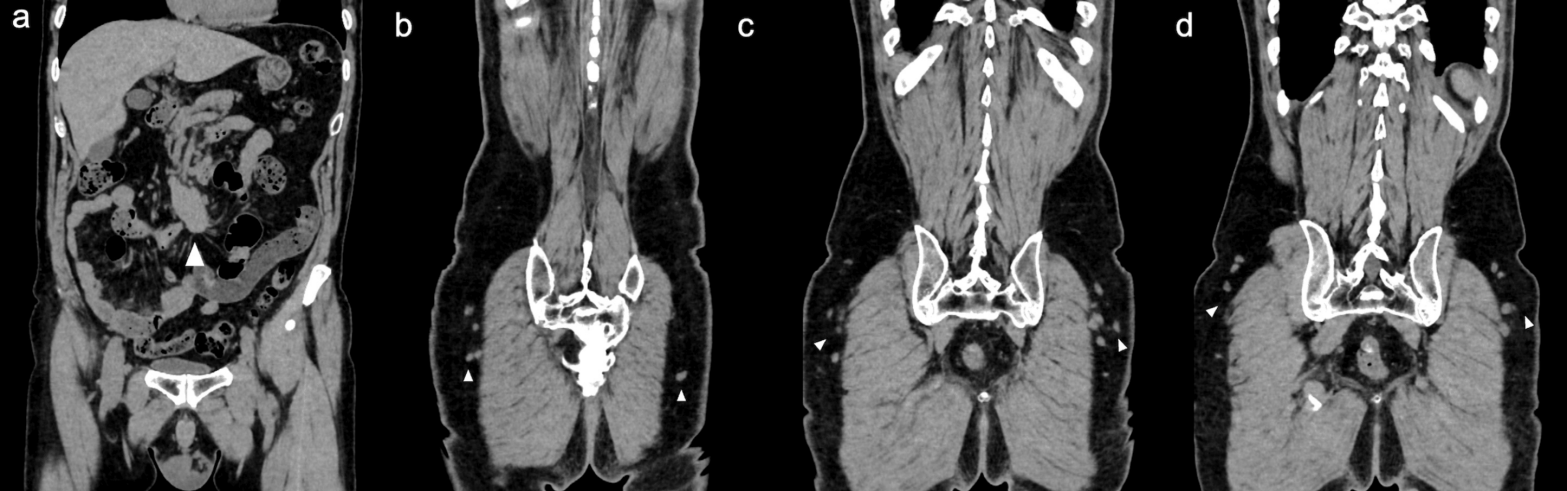
Contrast-enhanced CT images Contrast-enhanced CT images six years after treatment initiation. Coronal view showing no significant changes in primary lesion size (a: arrow) or new lesion development (a). Sequential coronal section images showing persistent lanreotide depots in the buttocks (b, c, d). Persistent nodular lesions (b, c, d: arrows) in subcutaneous nodules on the buttocks over time. CT, computed tomography

As of December 2024, the patient had received 72 doses of lanreotide, with no progression of the primary lesion (Figure 5). Given the stable disease status, lanreotide treatment will be continued until there is significant tumor progression.

## Discussion

Lanreotide is a widely used somatostatin analog that is used to suppress the progression of NETs [10,11], as in this case, as well as to treat hormone hypersecretion in other diseases, such as acromegaly [12] and TSH-producing pituitary adenoma [13]. Although the phenomenon of lanreotide depot remaining subcutaneously and mimicking metastatic lesions on imaging has been reported [14], it remains under-recognized. This case emphasizes the importance of educating healthcare providers on accurately interpreting imaging findings to avoid diagnostic errors.

Lanreotide depot is a sustained-release formulation designed through peptide self-assembly, forming a gel-like structure for prolonged drug release. This structure is composed solely of lanreotide acetate and water, without excipients, resulting in a highly organized hexagonal lattice of nanotubes. This unique feature not only ensures sustained drug delivery but also supports a four-week dosing interval, as demonstrated in pharmacokinetic studies [9]. However, this depot can persist subcutaneously and appear as nodular lesions on imaging, potentially mimicking a metastatic disease. The prolonged presence of the drug is attributed to the gradual diffusion of lanreotide from the depot into surrounding tissues as well as the stability of the nanotube assembly in vivo [9].

CT imaging may reveal these subcutaneous depots as well-defined nodular densities in the subcutaneous tissue. Understanding the specific characteristics of these lesions, such as their well-defined shape, stable position over time, and prolonged persistence, is essential for differentiating them from metastatic lesions. Previous reports, including the present case, emphasize that recognizing these imaging features can help avoid unnecessary diagnostic procedures and misinterpretations [15]. For example, a retrospective study involving 56 patients with metastatic midgut carcinoid tumors demonstrated that 67% of patients receiving somatostatin analogs developed subcutaneous nodules at injection sites, whereas no nodules were detected in patients who did not receive this therapy [15].

A previous study indicated a higher incidence of subcutaneous nodules in lanreotide users (73%) than in octreotide users (42%) due to injection techniques (subcutaneous vs. intramuscular) [15]. Understanding these differences in depot formation is crucial for distinguishing between benign lanreotide depots and true metastatic lesions. These nodules were typically small (mean size: 1 cm) and multiple, often located near the surface for lanreotide and deeper for octreotide, likely due to differences in injection techniques (subcutaneous vs. intramuscular). Importantly, these nodules are generally asymptomatic and tend to regress over time without clinical consequences, which can reassure both patients and clinicians when interpreting the CT results. Additionally, the absence of nodules in areas unrelated to injections further supports the localized nature of this phenomenon rather than indicating systemic metastatic progression. Recognizing these nodules as lanreotide depots can prevent unnecessary biopsies and reduce patient anxiety, especially during long-term NET management.

Finally, lanreotide plays a critical role in NET management, as demonstrated in clinical trials. The CLARINET trial demonstrated significant prolongation of progression-free survival in patients with grade 1 or 2 enteropancreatic NETs (Ki-67 < 10%) [7,8], establishing lanreotide as a cornerstone therapy for NETs, irrespective of hepatic tumor volume or baseline disease stability. Given its broad applications and critical role in both hormonal symptom control and tumor progression management, raising awareness of its unique imaging implications is crucial.

## Conclusions

This case highlights the critical need for increased awareness of lanreotide depot retention as a potential imaging artifact that can mimic metastatic disease. While lanreotide remains a cornerstone in NET management due to its ability to prolong progression-free survival, its radiological appearance should be carefully evaluated to prevent misdiagnosis and unnecessary interventions. Physicians, radiologists, and oncologists should be familiar with the characteristic imaging findings of persistent lanreotide depots to avoid unnecessary concerns regarding disease progression.
